# General, 21-Day Postoperative Rehabilitation Program Has Beneficial Effect on Oxidative Stress Markers in Patients after Total Hip or Knee Replacement

**DOI:** 10.1155/2020/4598437

**Published:** 2020-09-24

**Authors:** Bronisława Skrzep-Poloczek, Jakub Poloczek, Elżbieta Chełmecka, Wojciech Kazura, Agnieszka Dulska, Maciej Idzik, Jerzy Jochem, Dominika Stygar

**Affiliations:** ^1^Department of Physiology, Faculty of Medical Sciences in Zabrze, Jordana Street 19, 41-808 Zabrze, Medical University of Silesia, Katowice, Poland; ^2^Department of Rehabilitation, 3rd Specialist Hospital in Rybnik, Energetyków 46 Street, 44-200 Rybnik, Poland; ^3^Department of Statistics, Department of Instrumental Analysis, Faculty of Pharmaceutical Sciences in Sosnowiec, Ostrogórska 31 Street, 41-200 Sosnowiec, Medical University of Silesia, Katowice, Poland; ^4^Department of Pregnancy Pathology, Department of Woman's Health, School of Health Sciences in Katowice, Stefana Batorego 18, 41-902, Bytom, Medical University of Silesia, Katowice, Poland; ^5^Independent Public Health Care, Opole Cancer Center prof. Tadeusz Koszarowski, Katowicka 45-061 Street, 46-020 Opole, Poland

## Abstract

Imbalance in prooxidant-antioxidant equilibrium plays an important role in the progression of osteoarthritis (OA). Postoperative rehabilitation significantly improves the functional activity of patients with OA. We aimed to assess the effect of the general 21-day postoperative rehabilitation on the oxidative stress markers in patients after total hip arthroplasty or knee replacement. Patients (n =41) started individually designed postoperative rehabilitation ca. 90 days after endoprosthesis implantation. We used the six-minute walk test (6MWT) to quantify the changes in their exercise capacity. We analyzed the oxidative stress markers: total antioxidant capacity (TAC), total superoxide dismutase (SOD), Cu-Zn-superoxide dismutase (CuZnSOD) and ceruloplasmin (Cp) activity, malondialdehyde (MDA) and lipofuscin (LPS) concentration in patients serum to asses changes in the oxidative stress intensity. We found that after 21-days postoperative rehabilitation program: the average distance walked by patients increased by 69 m; TAC increased by 0.20 ± 0.14 mmol/l; both SOD isoforms activities increased by 1.6 (±1.7) and 1.72 (±1.5) NU/ml, respectively; but Cp activity decreased by 1.8 (0.7-3.7) mg/dl. Also, we observed lower concentrations of lipid peroxidation markers: by 19.6 ± 24.4 *μ*mol/l for MDA and by 0.4 ± 0.5 RF for LPS. A 21-day postoperative rehabilitation program effectively reduces oxidative processes, which helps the patients after total hip or knee replacement in a successful recovery.

## 1. Introduction

Knee and hip osteoarthritis (OA) are frequent conditions in elder individuals, as osteoarthritis itself is one of the most common joint disorders in the world [1]. Pain and disability, two most frequent OA complications, are prevalent among middle-aged and elderly individuals [2]. Total hip arthroplasty and knee replacement performed in patients with end-stage arthritis become more frequent since the population is aging. The effective procedure relieves patients from pain, facilitates functional activities and promotes their return to the daily activities [3, 4]. The positive role of general rehabilitation in OA has been confirmed in numerous studies. Rehabilitation, regardless of its type, significantly reduces pain, increases range of motions and muscle strength, and reduces the use of medications [5–7]. By some clinical practitioners, it is even adapted as preparation for the arthroplasty procedure [8, 9]. However, no scientific report specifies what type of rehabilitation therapy is most optimal for the patient's recovery process after total hip arthroplasty and knee replacement and no routine rehabilitation protocol has been developed as of yet.

Osteoarthritis is a multifactorial process of joint degeneration and various mechanisms may underlay its development [10]. One of them is the disruption of pro-oxidants and antioxidants equilibrium, which promotes cellular oxidative stress and leads eventually to OA progression [11]. During exacerbated physical exercises, oxygen uptake and energy demand increase rapidly. This intensifies mitochondrial energy metabolism, which enhances free radicals formation [12, 13]. The intensity of oxidative stress can be assessed using various general and specific indicators [14–16]. Total antioxidant capacity (TAC) shows the interaction between synergistic and antagonistic antioxidants. Its measurements are essential to correctly assess the results of other, more specific, oxidative stress markers [17]. Superoxide dismutase (SOD) along with its copper-zinc isoform (CuZnSOD) is one of the most important detoxifying enzymes and indicators of oxidative stress and reactive oxygen species increase [7]. Ceruloplasmin (Cp), mainly described as the acute phase protein and copper carrier to tissues, is also a prominent antioxidant scavenging reactive oxygen species and having pleiotropic effects on the body's antioxidative and oxidative metabolism [18]. When oxidative stress exceeds the compensatory capabilities of the cells and SOD fails to scavenge free radicals effectively, lipid peroxidation and cell damage occur. MDA is a product of this reaction and may be successfully used as an indicator of the severity of the oxidative processes in cells. Given these facts, malondialdehyde (MDA) and SOD are considered matching oxidative stress markers [12, 13]. Lipofuscin is another product of the unsaturated fatty acids oxidation. It is closely related to the natural aging processes and its accumulation in the body manifests as pigment deposits in skin, liver, kidneys or heart muscle [19]. Lipofuscin accumulation in organs may result from the imbalance between its formation and its degradation in lysosomes, and the formation process is directly related to an increased concentration of other oxidative stress markers [19].

It is known that rehabilitation significantly improves functional activity and quality of life of patients with osteoarthritis. However, it remains mostly unknown how rehabilitation affects biochemical processes of the body, especially antioxidative processes at the cellular level. This study aimed to assess the effect of a general 21-day rehabilitation program on the oxidative stress markers in patients after total hip arthroplasty or knee replacement.

## 2. Materials and Methods

### 2.1. Ethical Statement and Permissions

The study was carried out according to the Declaration of Helsinki and the protocol was fully approved by the Ethics Committee of the Medical University of Silesia in Katowice (N° KNW/002/KB1/106/17; 03.10.2017). Every participant of the study received the description of the protocol and was informed about its benefits and possible risks and returned the written informed consent before the study started.

### 2.2. Study Group

Forty-one patients after total hip (n =29; 71%) or knee (n =12; 29%) replacement, aged 61.0 ± 8.1 years, were included in the study. Twenty-two of them were men and 19 were women (54% and 46%, respectively) and on the initial examination day, they were, on average, 89.6 ± 9.7 days after the replacement surgery. Due to concomitant health conditions that occurred during the study, results of five patients were excluded from the oxidative stress markers analyses. Further details are presented in section 3.4.

Upon the arrival to the outpatients' clinic, the resting electrocardiogram (ECG) and blood pressure measurement were recorded, the body mass and height measurements were taken for each patient. Also in each case, the clinical interview was carried out to exclude patients with inflammatory disorders, infections, renal or hepatic insufficiency, active coronary artery disease, diabetes, heart failure, hormonal replacement therapy or supplementation with antioxidants that might have occurred or taken place 3 months before the study.

### 2.3. General Rehabilitation Program

All patients underwent a 21-day general rehabilitation program that started ca. 90 days after knee or hip endoprosthesis implantation. Rehabilitation sessions were conducted daily for 21 days, starting between 8 : 00 and 8 : 45 am. The main components of general rehabilitation program were physiotherapy, daily living activities training and patient's education on nutrition. The individual rehabilitation programs consisted of aerobic walking (30-45 min), strength training (20-30 min), rotor/bicycle training (30-45 min) and a cool-down phase (15 min). The patients were advised to continue the learned exercises and pro-health behaviour over the course of the day to keep their physical fitness and biochemical parameters at the beneficial level [20]. The programs were individually created for each patient, in regards to the choice of exercises (different strength and balance exercises) and training modalities (number and sets of repetitions as well as the duration of resting time), and then monitored in the rehabilitation by the responsible physiotherapist.

Osteoarthritis severely reduces the patient's exercise capacity. To quantify the exercise capacity, clinical practitioners use the 6-minute walk test (6MWT) [21]. In our study, each patient underwent the 6MWT twice: before starting and after completing the 21-day general rehabilitation program. The aim was to assess changes in functional exercise capacity and thus, to determine the effectiveness of the rehabilitation program [22, 23].

### 2.4. Samples Collection

Blood samples were collected before the initial and after the final rehabilitation session. Blood samples (5 mL) from ulnar vein were collected in the morning, at 8 : 00 AM, before breakfast. Blood was collected to the standard blood tubes: with EDTA (1.6 mg/ml EDTA-K_3_; S-Monovette, SARSTEDT) and into tubes with a clot activator (S-Monovette, SARSTEDT). The samples for serum analysis were centrifuged at 4000 rpm for 10 minutes at 4°C, and stored in –80°C. Plasma and serum samples were subsequently frozen and stored at −80°C until biochemical analyses could be performed.

#### 2.4.1. Oxidative Stress Marker Analysis

The state of the antioxidant system was analyzed in the serum samples. The enzymatic antioxidant markers were assessed by measuring total superoxide dismutase (SOD), copper-zinc-superoxide dismutase (CuZnSOD) and ceruloplasmin (Cp) concentration. Non-enzymatic antioxidant systems were analyzed by assessing total antioxidant capacity (TAC), lipofuscin (LPS) and malondialdehyde (MDA) concentration.

#### 2.4.2. Total Antioxidant Capacity (TAC)

TAC was measured using a commercial kit (Randox Co., England). The 2,2'-azino-di-(3-ethylbenzothiazoline sulphonate) (ABTS) was incubated with a peroxidase (metmyoglobin) and hydrogen peroxide to produce the radical cation ABTS+, which has a relatively stable blue-green color and can be measured at 600 nm. The suppression of the color was compared to the standard for TAC measurement assays (Trolox). The results are expressed as Trolox equivalent (mmol/l). The inter- and intra-assay coefficients of variations (CV) were 1.1% and 3.8%, respectively.

#### 2.4.3. Superoxide Dismutase (SOD) Activity (EC 1.15.1.1)

SOD isoenzymes' activity was determined with the use of the spectrophotometric method by Oyanagui [23]. KCN was used as the inhibitor of the CuZnSOD isoenzyme. SOD activity was calculated against a blank probe (containing bidistilled water). Enzyme activity was expressed as nitrite units (NU) per mg of protein. One NU exhibits 50% inhibition of formation of nitrite ion under the method' s condition [24].

#### 2.4.4. Ceruloplasmin (Cp) Concentration

Ceruloplasmin (Cp) concentration was measured using the p-phenylenediamine kinetic method by Richterich [25] and expressed in mg/dl after calibration with pure ceruloplasmin isolated from a healthy donor serum pool. The inter- and intra-assay coefficients of variations (CV) were 3.1% and 6.1%, respectively.

#### 2.4.5. Malondialdehyde (MDA) Concentration

MDA concentration was measured using the spectrophotometric method (wavelengths: 552 nm for emission, 515 nm for excitation; Perkin Elmer LS45 spectrofluorimeter by Ohkawa et al. [26]. and standard curve prepared for 1,1,3,3-tetraethoxypropane - the product of malondialdehyde and thiobarbituric acid reaction. MDA concentration was expressed in (*μ*mol/l).

#### 2.4.6. Lipofuscin (LPS) Concentration

LPS concentration was determined according to Tsuchida et al. [27]. Serum was mixed with ethanol-ether (3 : 1, v/v), shaken and centrifuged. The intensity of fluorescence was determined using a PerkinElmer spectrophotometer LS45 at a wavelength of 345 nm (absorbance) and 430 nm (emission) in a dissolved solid. The values are expressed in relative lipid extract fluorescence (RF), where 100 RF corresponds to the fluorescence of 0.1 *μ*g/ml quinidine sulphate in 0.1 N sulfuric acid. LPS concentrations are shown in RF. The inter- and intra-assay coefficients of variations (CV) were 2.8% and 9.7%, respectively.

### 2.5. Statistical Analysis

Distribution of variables was evaluated by the Shapiro-Wilk test and quantile-quantile plot. The interval data were expressed as a mean value ± standard deviation (M ± SD) in the case of normal data distribution or as a median (lower – upper quartiles; Me (Q1-Q3)) in the case of skewed or non-normal data distribution. The t-Student's test for dependent variables or non-parametric Wilcoxon's test were used for data comparison. In the case of skewed data distribution, the logarithmic transformation was done before analysis. To assess the relationship between qualitative variables, the *χ*^2^ test was used. Statistical significance was set at a *p* <0.05 and all tests were two-tailed. Statistical analysis was performed using data analysis software system Statistica, version 13.3.0 (TIBCO Software Inc., USA).

## 3. Results

In our study groups we observed interesting statistical tendency. Hip arthritis was more frequent in male patients, while knee arthritis was more frequent in female patients, but those results were not statistically significant (*χ*2 = 2.81, *p* =0.093, Table 1).

### 3.1. General Health Indicators

Biochemical and morphological characteristics of the blood of patients before and after the 21-day general rehabilitation program are presented in Table 2. Our results show that the individually designed general rehabilitation had positive effects on the patients' blood glucose and lipids concentrations: glucose, total cholesterol, LDL, and TG were significantly lower and HDL levels were significantly higher when compared to their initial levels. Also, we observed that CRP, platelets and haematocrit were lower after the rehabilitation what proves that general rehabilitation helps to reduce inflammation and prevents from clot formation.

### 3.2. Body Mass

The 21-day general rehabilitation program did not influence the patients' body mass. Their body mass in the pre-rehabilitation period was 86.1 (± 11.7) kg on average and was the same as in the post-rehabilitation period (84.4 ± 11.6 kg) (*p* = 0.546; Table 3).

### 3.3. Six-Minute Walk Test (6MWT)

**Figure 1:**
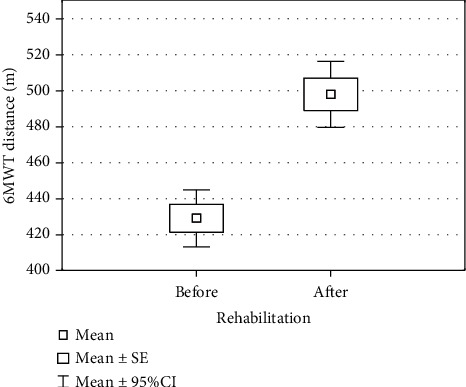
Results of the 6-minute walk test distance (6MWT) (m) of the patients (n =41) with hip or knee endoprosthesis before and after a 21-day general rehabilitation program. Symbols used in the plot: inner box – mean value, outer box – mean value ± standard error, whiskers – mean value ±95% confidence interval.

The 21-day general rehabilitation program improved patient's walking capacity by 69 m. The average distance walked by patients before rehabilitation program was 428.9 (± 46.8) m, whereas after rehabilitation they were able to cover the distance of 497.8 (± 54.2) m. The increase in the results of 6MWT was statistically significant (*p* <0.001) (Table 3, Figure 1).

### 3.4. Oxidative Stress Markers

Since five patients were excluded from the study due to concomitant health conditions, the results of oxidative stress markers were obtained for 36 patients: 18 men and 18 women, aged 58.8 ± 8 years. 24 of them were after total hip (66.6%) and 12 of them were after knee (33.3%) replacement surgery performed 89 ± 2 days earlier.

We observed an increase in total antioxidant capacity (TAC) by about 0.20 ± 0.14 mmol/l (95% PU: 0.15-0.25) (*p* <0.001; Table 4; Figure S1 in Supplementary Materials) and in the activities analyzed of superoxide dismutase (SOD) isoforms. Total SOD activity was on average 1.6 ±1.7 NU/ml (95% PU: 1.1-2.2) (*p* <0.001; Table 4; Figure S2 in Supplementary Materials) higher and, similarly, CuZnSOD activity was on average 1.7 ±1.5 NU/ml (95% PU: 1.2-2.2) (*p* <0.001; Table 4; Figure S3 in Supplementary Materials) higher in the serum of patients after 21-day rehabilitation program when compared to their activities before starting the program. On the contrary, a 21-day general rehabilitation reduced ceruloplasmin (Cp) levels in patients' serum by 1.8 (0.7-3.7) mg/dl (*p* < 0.001; Table 4; Figure S4 in Supplementary Materials).

We observed significant decrease, by 19.6 ± 24.4 *μ*mol/l (95% PU: 11.4-27.9), in MDA concentration in blood of patients after a 21-day rehabilitation program (*p* <0.001; Table 4; Figure S5 in Supplementary Materials). A similar effect of the rehabilitation program we observed for LPS concentration in the blood of patients after hip or knee replacement: its concentration in the post-rehabilitation period decreased by 0.4 ± 0.5 RF units (95%PU: 0.2-0.5) when compared to the period before rehabilitation (*p* < 0.001; Table 4, Figure S6 in Supplementary Materials).

## 4. Discussion

In this study, we assessed the impact of postoperative rehabilitation on antioxidant stress markers in patients after hip or knee replacement surgery. We analyzed the total antioxidant capacity (TAC), the activity of total superoxide dismutase (SOD), Zn-Cu-superoxide dismutase (ZnCuSOD) and ceruloplasmin (Cp) concentration, as well as the concentration of malondialdehyde (MDA) and lipofuscin (LPS) – the oxidative stress markers – in patients subjected to general rehabilitation after knee or hip endoprosthesis implantation. To the best of our knowledge, this is the first study designed to compare the oxidative stress markers before and after the general rehabilitation cycle. Here, we report a significant increase in TAC level, SOD and ZnCuSOD activities and simultaneous reduction in Cp, LPS and MDA plasma levels after the 21-day general rehabilitation program. The physical rehabilitation not only improved all oxidative stress parameters but also positively affected patients' general health, as indicated by glucose and lipids profiles and inflammation and blood clotting parameters. It also significantly improved patients' physical efficiency and exercise capacity determined by the 6-minute walk test (6MWT). Although the walked distance in 6MWT has increased after the rehabilitation process, the patients' body mass remained the same, as expected.

### 4.1. Total Antioxidant Capacity (TAC)

Osteoarthritis affects patients' antioxidative and oxidative metabolism by significantly impairing antioxidant defense [28]. A decrease in total antioxidant capacity results from enhanced oxidative stress, making patient's body more susceptible to damage caused by the increased amount of reactive oxygen species [29].

We found that the 21-day rehabilitation program conducted after the endoprosthesis implantation surgery significantly enhanced total antioxidant capacity. Our results coincide with those obtained by Porter et al. [30] analyzing the effects of long-term aerobic training on TAC of the human body [30]. The study design analysis showed that the type of physical exercises from this study corresponds to exercises included in our rehabilitation program. Therefore our results support findings of others, that, not intense but rather long and regular physical effort contributes to the improvement of redox balance [7, 31]. Stanek et al. [32] observed a significant increase in TAC in patients with ankylosing spondylitis that were subjected to a series of whole-body cryotherapy followed by a subsequent kinesiotherapy. In this case, the combination of whole-body cryotherapy and kinesiotherapy was more effective that the kinesiotherapy itself. The authors concluded that their approach to ankylosing spondylitis treatment helped to decrease the oxidative stress ocurring during the active phase of this chronic inflammatory rheumatic disease [32].

### 4.2. Total Superoxide Dismutase (SOD) and cu-Zn Superoxide Dismutase (CuZnSOD)

In our study, 21-day general rehabilitation program resulted in increased superoxide dismutase (SOD) and its Cu-Zn isoform (CuZnSOD) activity. According to two independent studies, by Scott et al. [33] and Olszewska-Słonina et al. [34], total SOD activity significantly depletes in patients with osteoarthritis. The latter study, by Olszewska-Słonina et al. [34], proved positive impact of endoprosthesis surgery on total SOD plasma concentration, but the influence of postoperative rehabilitation or exercise was not considered in this study [34]. It was, however, the subject of numerous studies showing that SOD activity in skeletal muscles increases after the exercise [35–38]. Paans et al. [31] observed that the increase in total SOD activity is much more significant in terms of duration than the intensity of physical training, what was supported by Ha et al. [7]. This research, on hatha yoga effect on oxidative stress, showed that total SOD activity increased better in response to exercise duration rather than to exercise intensity [7]. All of the above-mentioned studies contain exercise element that agrees with our study design and the rehabilitation protocol used by us.

Our results show that the implementation of the rehabilitation program shortly after the surgery might lead to increase of total SOD activity. Due to the methodology of our research, we had limited insight into the postoperative period only, not into the dynamics of the change. Nevertheless, the 21-day general rehabilitation, tailored to the patient's abilities, designed and controlled by a physiotherapist, resulted in a significant increase both in total SOD and CuZnSOD activity, what was compatible with results obtained by other research groups.

### 4.3. Ceruloplasmin (Cp)

In this study, we analyzed the ceruloplasmin concentration as an oxidative stress marker in patients who underwent endoprosthesis surgery and post-operative rehabilitation due to osteoarthritis. According to El-Barbary et al. [39], high levels of oxidative stress occurring in the course of rheumatoid arthritis and osteoarthritis may lead to observed elevated plasma Cp concentration [39]. On the contrary, Kudriavtseva et al. [40] reported a decrease in Cp levels in the course of osteoarthritis, but this could have resulted from extremely high oxidative stress caused by severe tissue damage, having lead even to the depletion of the selected antioxidant systems. In this case, the inefficient redox system could not match Cp production, which manifested as a decrease in its level [40]. Our results did not indicate the occurrence of a similar phenomenon in any of the patients. Therefore, the decreased level of Cp after the 21-day rehabilitation program may be a good indicator of oxidative stress reduction after the rehabilitation process. By looking at the changes in ceruloplasmin levels, we argue that rehabilitation has a positive impact on patient body redox status.

### 4.4. Malondialdehyde (MDA)

During exacerbated physical exercises, oxygen uptake and energy demand increase rapidly, which leads to the intensification of mitochondrial energy metabolism and, as a result, promotes free radicals formation which, if not scavenged effectively by SOD, cause lipid peroxidation and its by-product, malondialdehyde (MDA), formation. One of the main mechanisms of osteoarthritis pathogenesis is oxidative stress promoted by enhanced free radical production and therefore, not surprisingly, increased MDA levels are observed in the serum of the patients suffering from this condition [12, 13]. Many studies have shown that high-intensity exercise increases oxidative stress and intensifies lipid peroxidation processes [41–43], which is mainly the result of muscle damage and fatigue induced by exercise [43] However, the effect of physical activity on lipid peroxidation intensity is not completely known as it may be affected by many variables such as exercise type, intensity and duration [44, 45]. According to Dixon et al. [46], it is the intensity of resistance exercise bout that plays the main role in oxidative stress generation [46]. Also, it was found that MDA concentration, a by-product of the polyunsaturated fatty acid oxidation, increased during aerobic exercise [42, 43, 47]. On the other hand, it was proved that long-term participation in exercise can strengthen antioxidant defense, change beneficially redox status and reduce lipid peroxidation intensity and therefore their products formation [48–50]. Ha et al. [7] proved that 16 weeks of hatha yoga training contributed to a statistically significant reduction in MDA concentration in blood serum [7]. Moreover, Vincent et al. [50] showed that six months of low-intensity resistance exercise training, following an acute bout of aerobic exercise, lowered levels of lipid peroxidation in the serum of studied patients [50]. Stanek et al. [51] reported that decrease in antioxidant status played an important role in the pathogenesis of ankylosing spondylitis [51].

This may be due to a fact, that long-term resistance training causes qualitative and quantitative changes in respiration processes of skeletal muscle mitochondria, increasing thus antioxidant capacity [30].

In our study, we observed that MDA, the lipid peroxidation by-product, levels dropped down significantly in convalescents after the 21-day general rehabilitation. We conclude that this is the effect of the study design: the patients underwent regular, daily low-intensity physical exercises, without prolonged or acute bouts of exercise, that were individually tailored to their abilities. This might have changed the respiration processes in skeletal muscles at the mitochondrial level, as discussed above, and have increased patients' antioxidant capacity, manifested also by elevated TAC levels we observed in our study.

### 4.5. Lipofuscin (LPS)

Lipofuscin is a product of the unsaturated fatty acids oxidation and its formation is directly related to an increased concentration of oxidative stress markers. Free oxygen radicals damage mitochondria and other cellular organelles, leading eventually to the formation of non-biodegradable lipofuscin that is stored in lysosomes, hindering their phagocytic activity and ability to degrade not-efficient and damaged mitochondria. This phenomenon is inevitably associated with the aging process [52–54]. To the best of our knowledge, there are barely any studies linking lipofuscin concentration and osteoarthritis in humans. Moreover, this subject was never analyzed in the context of lipofuscin serum level and its changes after rehabilitation. In our study, lipofuscin concentration significantly decreased after the 21-day general rehabilitation program. We assume that initially high lipofuscin concentration was associated with high oxidative stress resulting both from previous joint damage and recent surgery. The rehabilitation program had a positive effect on reducing oxidative stress in our patients and contributed to the subsequent reduction of lipofuscin concentration, as expected from literature analysis [55].

## 5. Conclusions

We can conclude that the 21-day postoperative general rehabilitation program has a significant impact on balancing oxidative processes and significant reduction of oxidative stress markers in patients with hip or knee replacement. Individually tailored, systematic physical effort is a crucial element of the postoperative protocol, which helps patients to recover effectively after the surgery by improving the redox balance.

## Figures and Tables

**Table 1:** Endoprosthesis implantation frequency in the studied group according to surgery type and gender. Statistical significance was set at *p* <0.05.

Surgery type	Men n = 22	Women n = 19	*χ* ^2^	*p*
Hip	18 (82%)	11 (58%)	2.81	0.093
Knee	4 (18%)	8 (42%)		

**Table 2:** Comparison of blood parameters of the patients with hip or knee endoprosthesis before and after a 21-day general rehabilitation program. The results (n =41) are presented as median (lower-upper quartile) or mean ± SD. Statistical significance was set at *p* < 0.05.

Parameter	Before	After	*Δ* = before-after	t	*p*
CRP (mg/l)	1.8 (1.3-2.3)	1.5 (1.1-2.0)	0.2 (0.1-0.3)	3.59	**<0.001**
Glucose (mmol/l)	5.2 ± 0.6	4.9 ± 0.6	0.2 ± 0.3	5.72	**<0.001**
ESR (mm/l)	9.0 (7.0-13.0)	9.0 (7.0-11.0)	—	1.87	0.069
Serum creatinine (*μ*mol/l)	80.2 ± 10.3	81.9 ± 9.5	—	1.64	0.109
Platelets (K/*μ*l)	226.5 ± 53.2	217.4 ± 35.1	9.1 ± 26.3	2.15	**< 0.05**
Haematocrit (%)	41.3 ± 3.3	40.2 ± 2.7	1.1 ± 2.0	3.37	**< 0.01**
RBC (M/*μ*l)	4.6 ± 0.4	4.6 ± 0.4	—	0.06	0.953
HGB (g/dl)	13.7 ± 1.1	13.8 ± 0.9	—	1.83	0.074
WBC (K/*μ*l)	6.7 ± 1.6	6.9 ± 1.2	—	1.22	0.230
Cholesterol (mg/dl)	230.6 ± 34.1	215.8 ± 29.9	14.9 ± 18.7	5.09	**<0.001**
HDL (mg/dl)	39.3 ± 4.1	40.6 ± 3.5	-1.3 ± 1.9	4.36	**<0.001**
TG (mg/dl)	154.9 (143.8-167.8)	145.9 (135.4-154.3)	11.6 (4.5-21.8)	3.05	**< 0.01**
LDL (mg/dl)	159.4 ± 31.5	145.8 ± 27.6	13.7 ± 18.7	4.66	**<0.001**

Abbreviations: CRP – C-reactive protein; ESR – erythrocyte sedimentation rate; HDL – high-density lipoprotein cholesterol; HGB – haemoglobin; LDL – low-density lipoprotein cholesterol; RBC – red blood cells; WBC – white blood cells; TG – triglycerides.

**Table 3:** Results of 6MWT and body mass of the patients (n =41) with hip or knee endoprosthesis before and after a 21-day general rehabilitation program. Statistical significance was set at *p* < 0.05.

General health indicator	Before	After	*Δ* = after-before	t	*p*
Body mass (kg)	86.1 ± 11.7	84.4 ± 11.6		0.607	0.546
6MWT (m)	428.9 ± 46.8	497.8 ± 54.2	68.9 ± 27.8	14.866	**<0.001**

Abbreviations: 6MWT – the 6-minute walk test.

**Table 4:** Comparison of oxidative stress markers measured in the serum of patients (*n* = 36) with hip or knee endoprosthesis before and after a 21-day general rehabilitation program. Statistical significance was set at *p* < 0.05.

Oxidative stress marker	Before	After	*Δ* = before - after	t	*p*
TAC (mmol/l)	1.07 ± 0.15	1.27 ± 0.17	-0.20 ± 0.14	8.427	**<0.001**
SOD (NU/ml)	10.1 ± 2.1	11.7 ± 2.1	-1.6 ±1.7	5.760	**<0.001**
CuZnSOD (NU/ml)	7.25 ±1.79	8.97 ±1.70	-1.72 ±1.50	6.859	**<0.001**
Cp (mg/dl)	27.5 (24.2-31.8)	25.4 (22.0-29.6)	1.8 (0.7-3.7)	4.813	**<0.001**
MDA (*μ*mol/l)	152.5 ± 27.9	132.9 ± 24.4	19.6 ± 24.4	4.823	**<0.001**
LPS (RF)	1.6 ± 0.4	1.3 ± 0.3	0.4 ± 0.5	4.14	**<0.001**

Abbreviations: Cp – ceruloplasmin; CuZnSOD – copper-zinc SOD; LPS (RF) – lipofuscin (radiofrequency); MDA – malondialdehyde; SOD – total superoxide dismutase; TAC – total antioxidative capacity.

## Data Availability

The original data are available after contact with the corresponding author.

## Supplementary Materials

Supplementary Materials This article is accompnied by Supplementary Materials which contain Figures with visualized data of oxidative stress markers levels in the serum of patients (n = 36) with hip or knee endoprosthesis before and after a 21-day general rehabilitation program.
