# Supplementary material for: General, 21-Day Postoperative Rehabilitation Program Has Beneficial Effect on Oxidative Stress Markers in Patients after Total Hip or Knee Replacement
Source: Oxid Med Cell Longev. 2020 Sep 24;2020:4598437. doi: 10.1155/2020/4598437 (PMC7532996; doi:10.1155/2020/4598437)
Supplement: Supplementary Materials — This article is accompnied by Supplementary Materials which contain Figures with visualized data of oxidative stress markers levels in the serum of patients (n = 36) with hip or knee endoprosthesis before and after a 21-day general rehabilitation program. [file 4598437.f1.pdf]

SUPPLEMENTARY MATERIALS

General, 21-day postoperative rehabilitation program has beneficial effect on oxidative stress markers in patients after total hip or knee replacement

Bronisława Skrzep-Poloczek,[1] Jakub Poloczek,[2] Elżbieta Chełmecka,[3] Wojciech Kazura,[1] Agnieszka Dulska,[4] Maciej Idzik,[5] Jerzy Jochem,[1] and Dominika Stygar[1]

[1] Department of Physiology, Faculty of Medical Sciences in Zabrze, Jordana Street 19, 41-808 Zabrze, Medical University of Silesia, Katowice, Poland.
[2] Department of Rehabilitation, 3rd Specialist Hospital in Rybnik, Energetyków 46 Street, 44-200 Rybnik, Poland.
[3] Department of Statistics, Department of Instrumental Analysis, Faculty of Pharmaceutical Sciences in Sosnowiec, Ostrogórska 31 Street, 41-200 Sosnowiec, Medical University of Silesia, Katowice, Poland.
[4] Department of Pregnancy Pathology, Department of Woman's Health, School of Health Sciences in Katowice, Stefana Batorego 18, 41-902, Bytom, Medical University of Silesia, Katowice, Poland.
[5] Independent Public Health Care, Opole Cancer Center prof. Tadeusz Koszarowski, Katowicka 45-061 Street, 46-020, Opole, Poland.

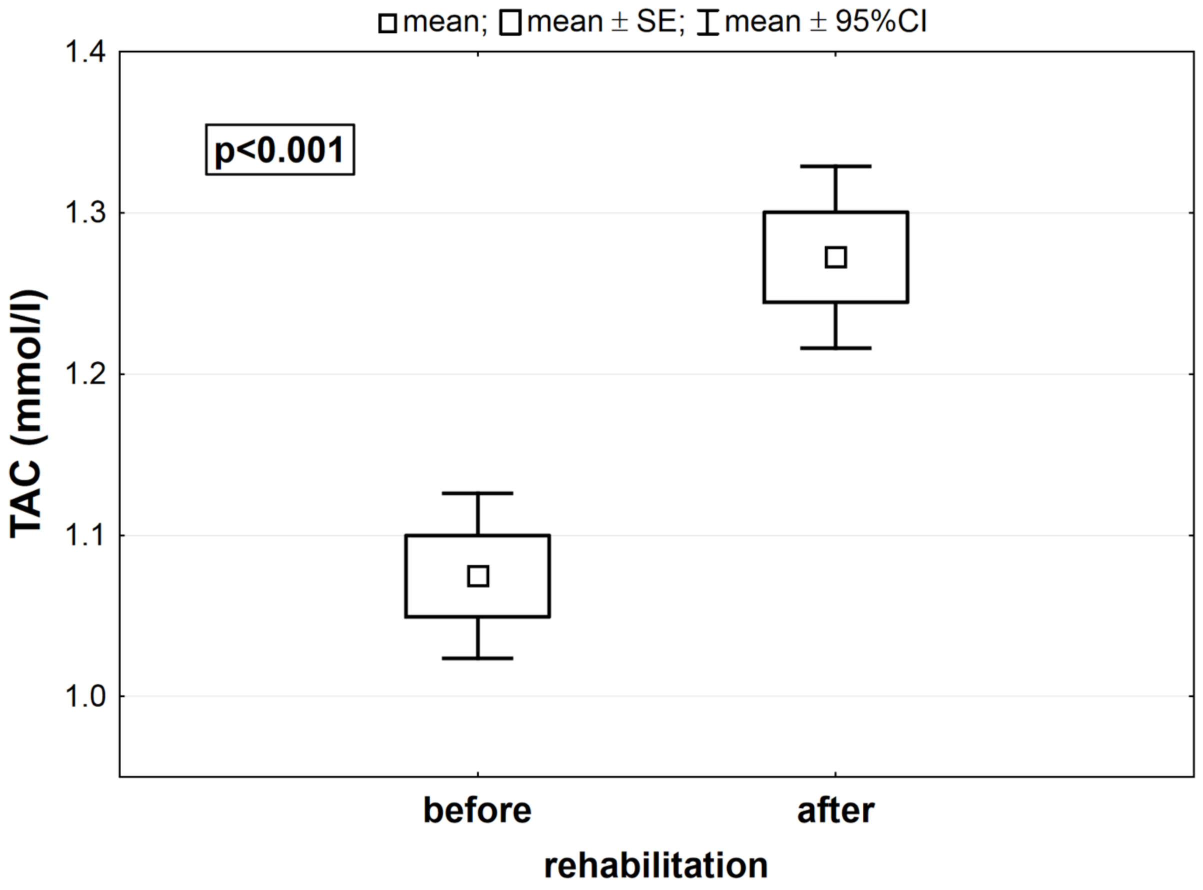

Figure S1. Total antioxidant capacity (TAC) (mmol/l) in the serum of patients (n =36) with hip or knee endoprosthesis before and after a 21-day general rehabilitation program. Symbols used in the plot: inner box – mean value, outer box – mean value ± standard error, whiskers – mean value ± confidence interval.

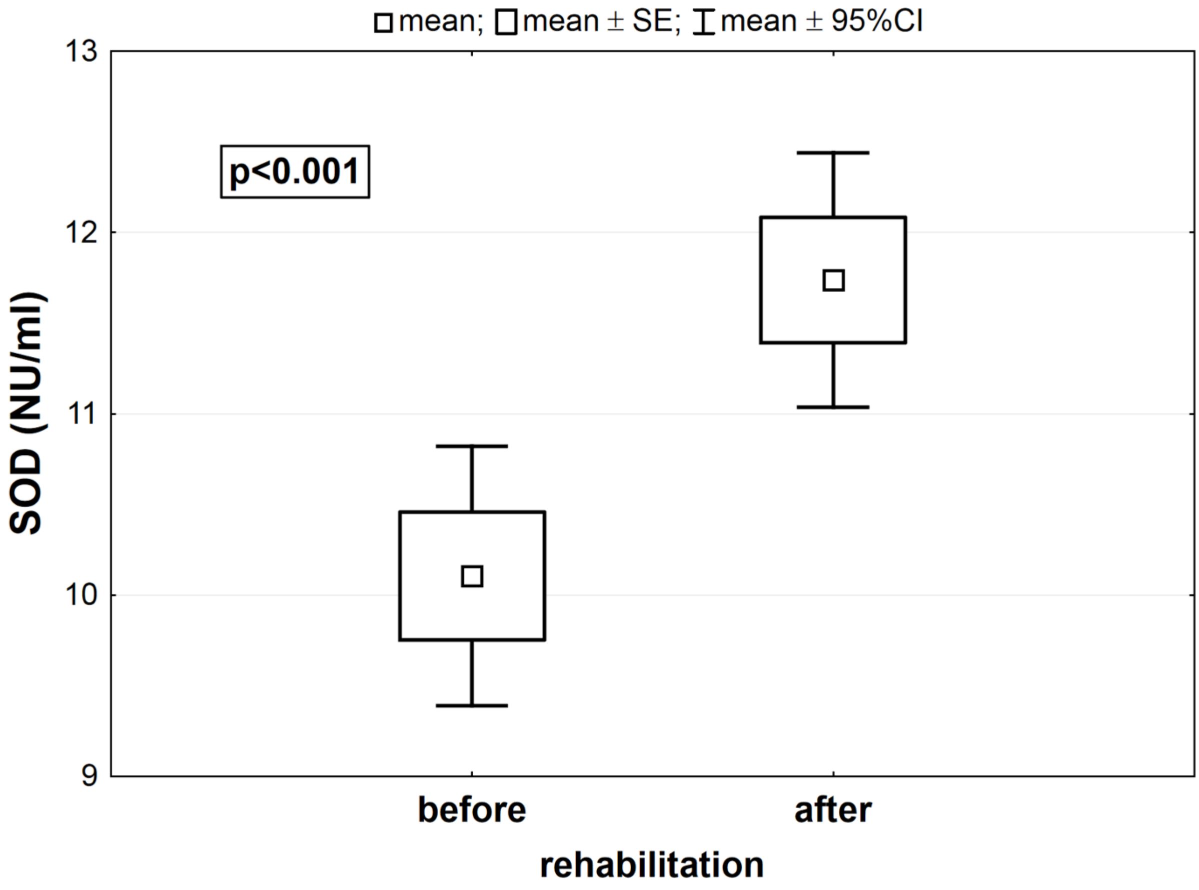

Figure S2. Total superoxide dismutase (SOD) activity (NU/ml) in the serum of patients (n = 36) with hip or knee endoprosthesis before and after a 21-day general rehabilitation program. Symbols used in the plot: inner box – mean value, outer box – mean value ± standard error, whiskers – mean value ± confidence interval.

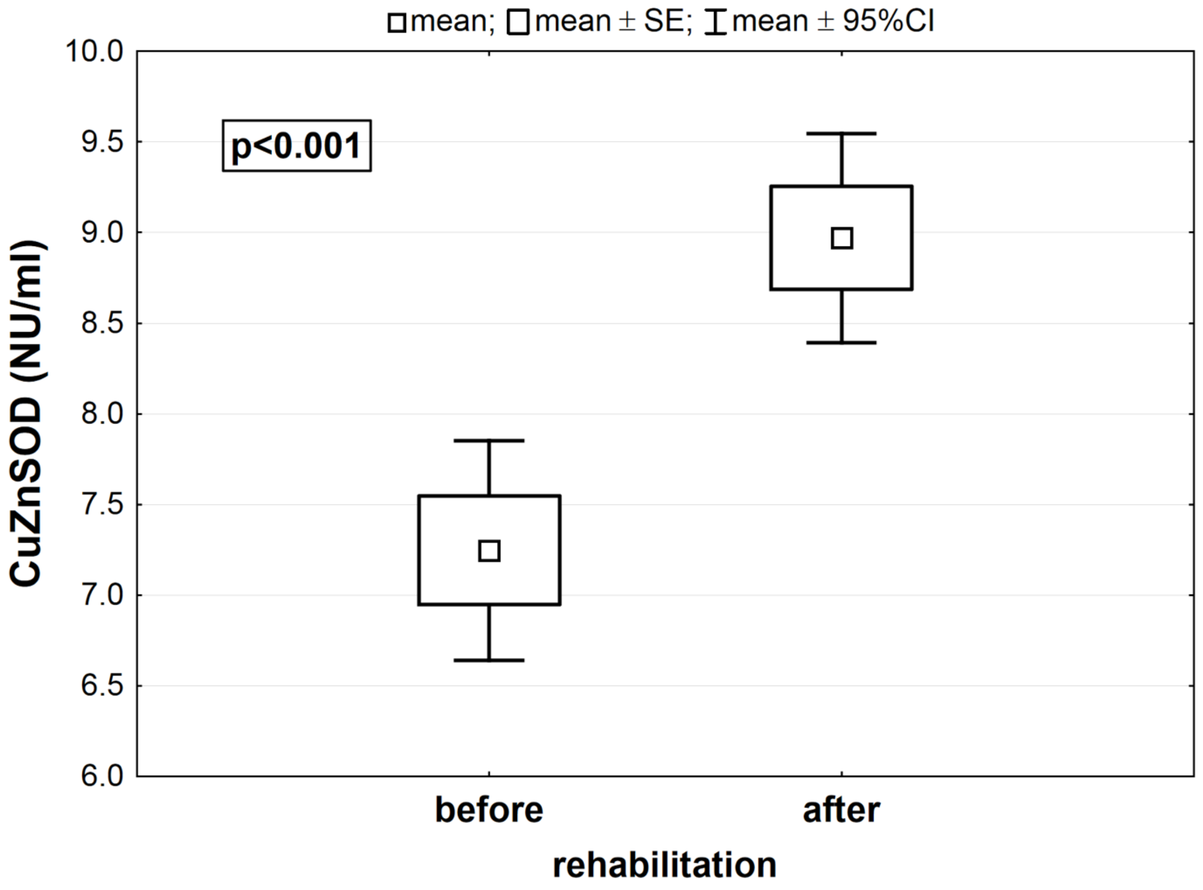

Figure S3. Cu-Zn superoxide dismutase (CuZnSOD) activity (NU/ml) in the serum of patients (n = 36) with hip or knee endoprosthesis before and after a 21-day general rehabilitation program. Symbols used in the plot: inner box – mean value, outer box – mean value ± standard error, whiskers – mean value ± confidence interval.

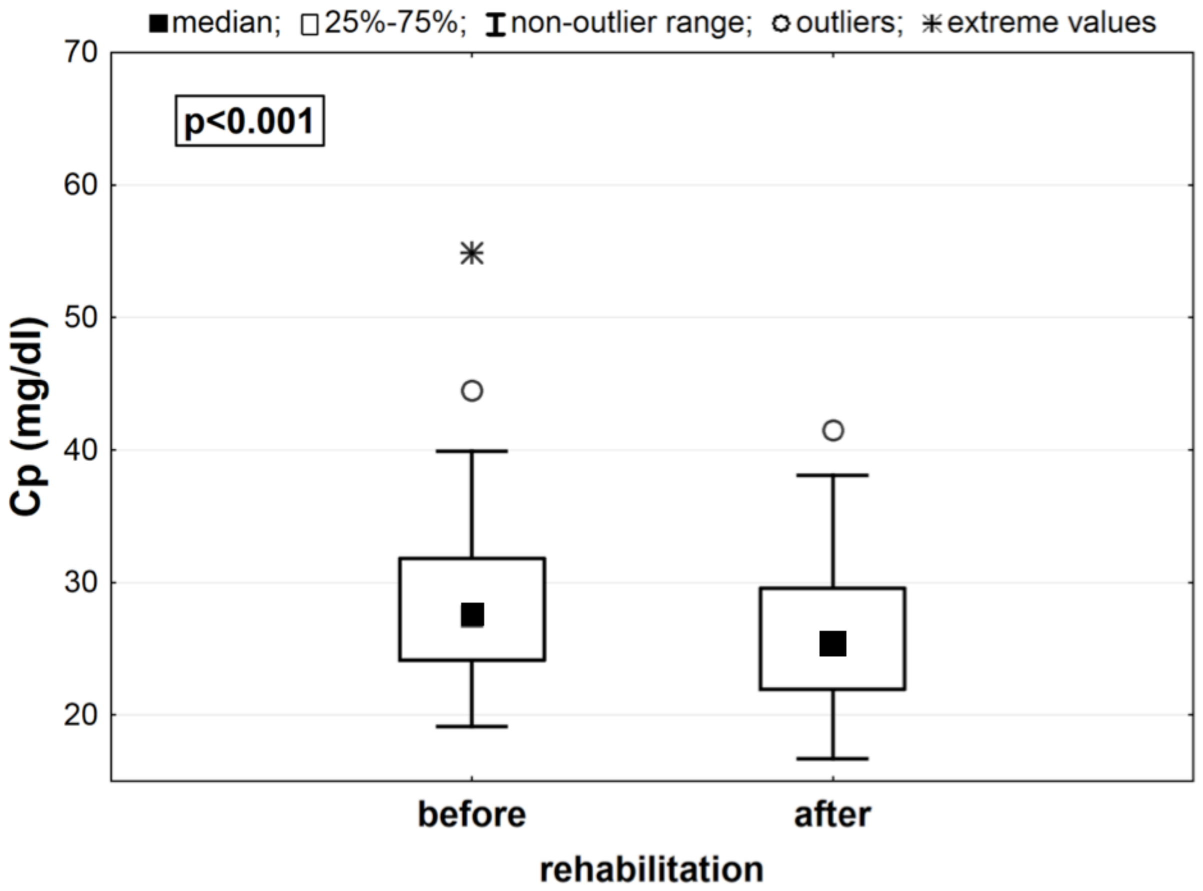

Figure S4. Ceruloplasmin (Cp) concentration (mg/ml) in the serum of patients (n = 36) with hip or knee endoprosthesis before and after a 21-day general rehabilitation program. Symbols used in the plot: inner box – median value, outer box – first quartile – third quartile, whiskers – non-outlier range, circle – outliers, * – extreme values.

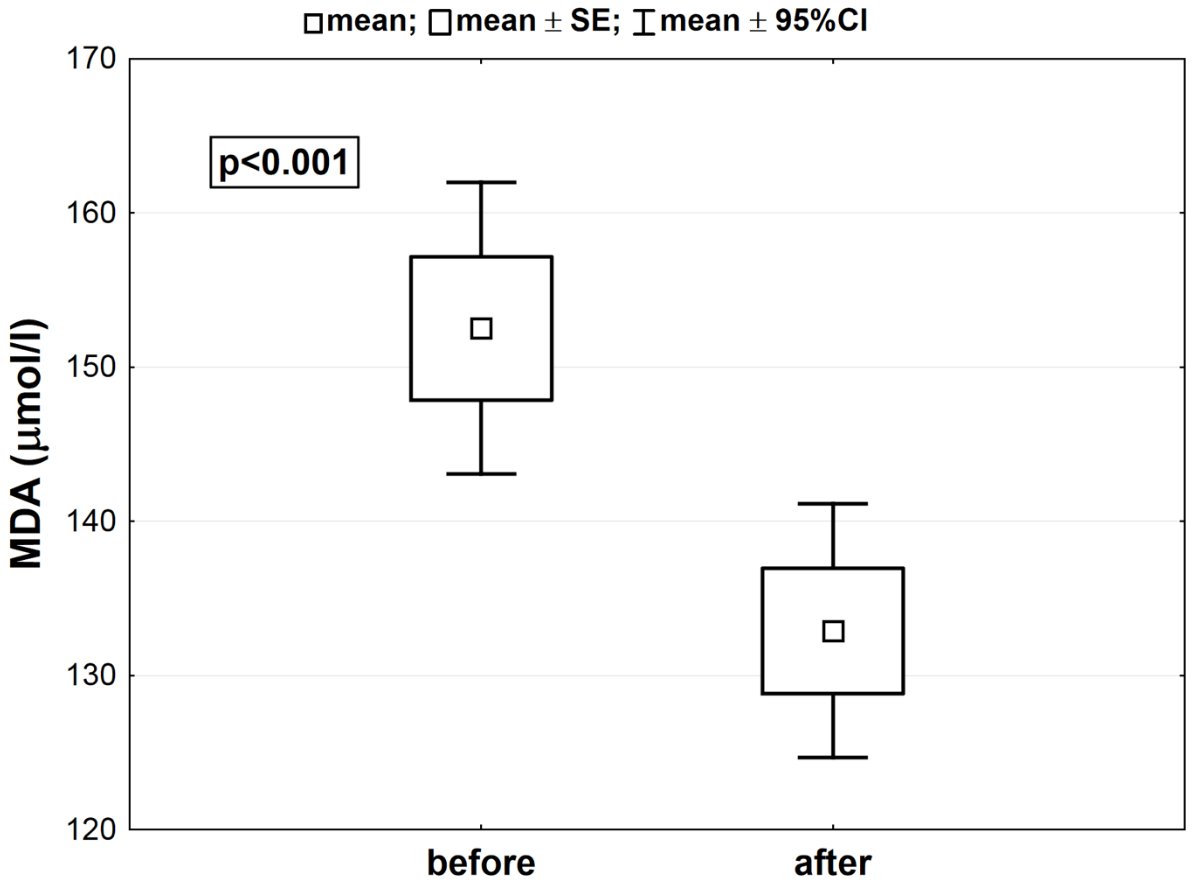

Figure S5. Malondialdehyde (MDA) concentration (µmol/l) in the serum of patients (n = 36) with hip or knee endoprosthesis before and after a 21-day general rehabilitation program. Symbols used in the plot: inner box – mean value, outer box – mean value ± standard error, whiskers – mean value ± confidence interval.

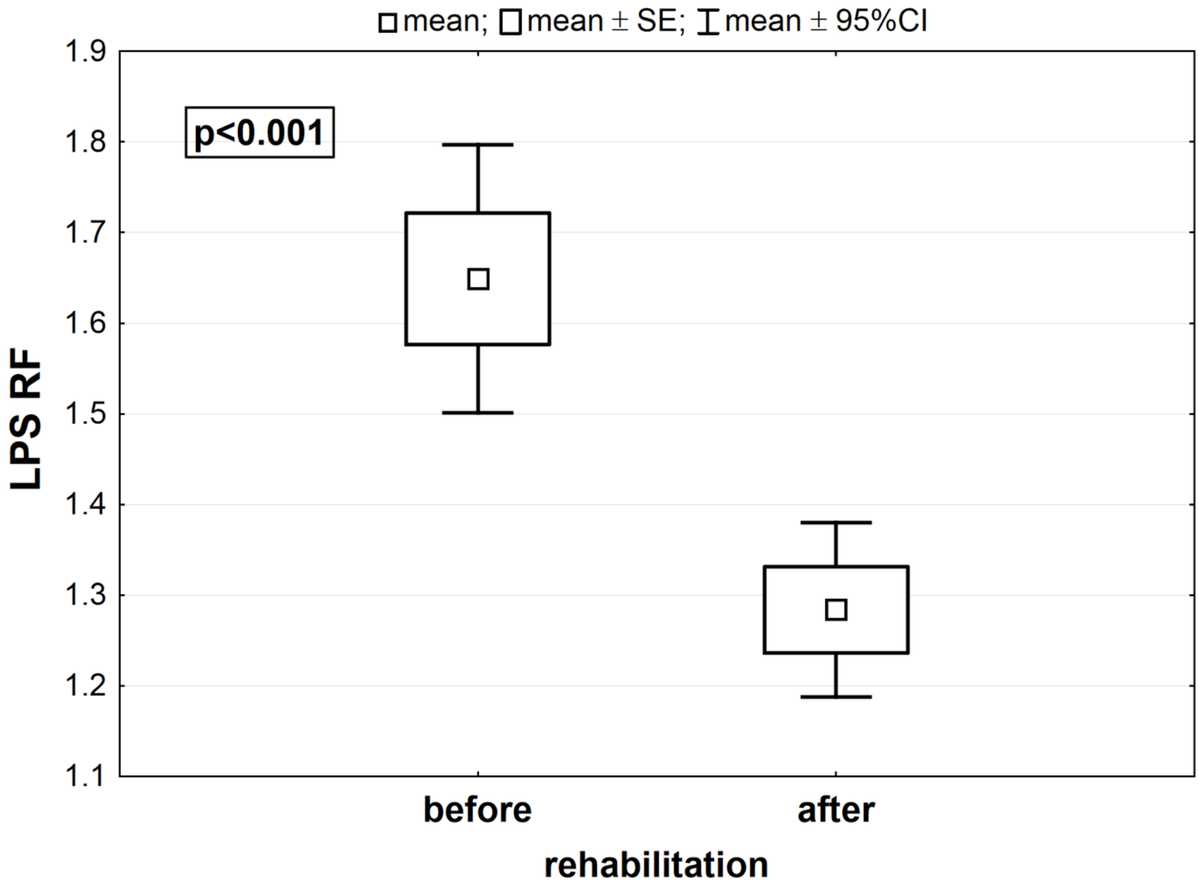

Figure S6. Lipofuscin (LPS) concentration (RF) in the serum of patients (n = 36) with hip or knee ednoprosthesis before and after a 21-day general rehabilitation program. Abbreviations: RF – radiofrequency. Symbols used in the plot: inner box – mean value, outer box – mean value ± standard error, whiskers – mean value ± confidence interval.
